# Mutational signatures: experimental design and analytical framework

**DOI:** 10.1186/s13059-020-1951-5

**Published:** 2020-02-14

**Authors:** Gene Koh, Xueqing Zou, Serena Nik-Zainal

**Affiliations:** 1 Wellcome Trust Sanger Institute, Wellcome Genome Campus, Hinxton, CB10 1SA UK; 2 Department of Medical Genetics, School of Clinical Medicine, University of Cambridge, Cambridge, CB2 0QQ UK; 3 MRC Cancer Unit, School of Clinical Medicine, University of Cambridge, Cambridge, CB2 0XZ UK

## Abstract

Mutational signatures provide a powerful alternative for understanding the pathophysiology of cancer. Currently, experimental efforts aimed at validating and understanding the etiologies of cancer-derived mutational signatures are underway. In this review, we highlight key aspects of mutational signature experimental design and describe the analytical framework. We suggest guidelines and quality control measures for handling whole-genome sequencing data for mutational signature analyses and discuss pitfalls in interpretation. We envision that improved next-generation sequencing technologies and molecular cell biology approaches will usher in the next generation of studies into the etiologies and mechanisms of mutational patterns uncovered in cancers.

## Introduction

Somatic mutations arising through cell-intrinsic and exogenous processes mark the genome with distinctive patterns termed mutational signatures. The field began in 2012 with the demonstration of at least 5 such mutation patterns in breast cancers [1]. Subsequently, 21 substitution signatures were identifiable across 30 cancer types [2]. While there have been revisions of analytical components of this field, there is a parallel trajectory evolving, focused on experimental validation, delineating aetiologies, and mechanisms of mutagenesis. This is important, as the field is quickly gaining traction in the clinical arena. To provide the required confidence that mutational signatures can be utilised clinically, it is necessary to cultivate supporting experimental evidence for mutational signatures to serve as potential biomarkers.

Several experimental studies to validate mutational signatures have been conducted, employing various model systems including *C. elegans*, yeast, human cancer cell lines, organoids, and human induced pluripotent stem cells among others [3–14]. There exist differences in how these studies were performed and how data were processed, analysed, and interpreted with different algorithms.

In this review, we present guidelines that we hope will facilitate future experiments and analyses. We focus on considerations in experimental design and on the computational framework for data analysis in mutational signature studies, particularly in human cellular model systems. We further discuss issues that need to be contemplated when linking an environmental mutagen or a DNA repair process to a mutational signature, which is not as straightforward as may superficially seem.

## Experimental considerations

### Choice of cellular model system

Three critical points require consideration when choosing a human cellular system for investigating mutagenesis: the average ploidy, its genetic background (cancer versus non-cancerous), and the likelihood of on-going mutagenesis.

**Fig. 1:**
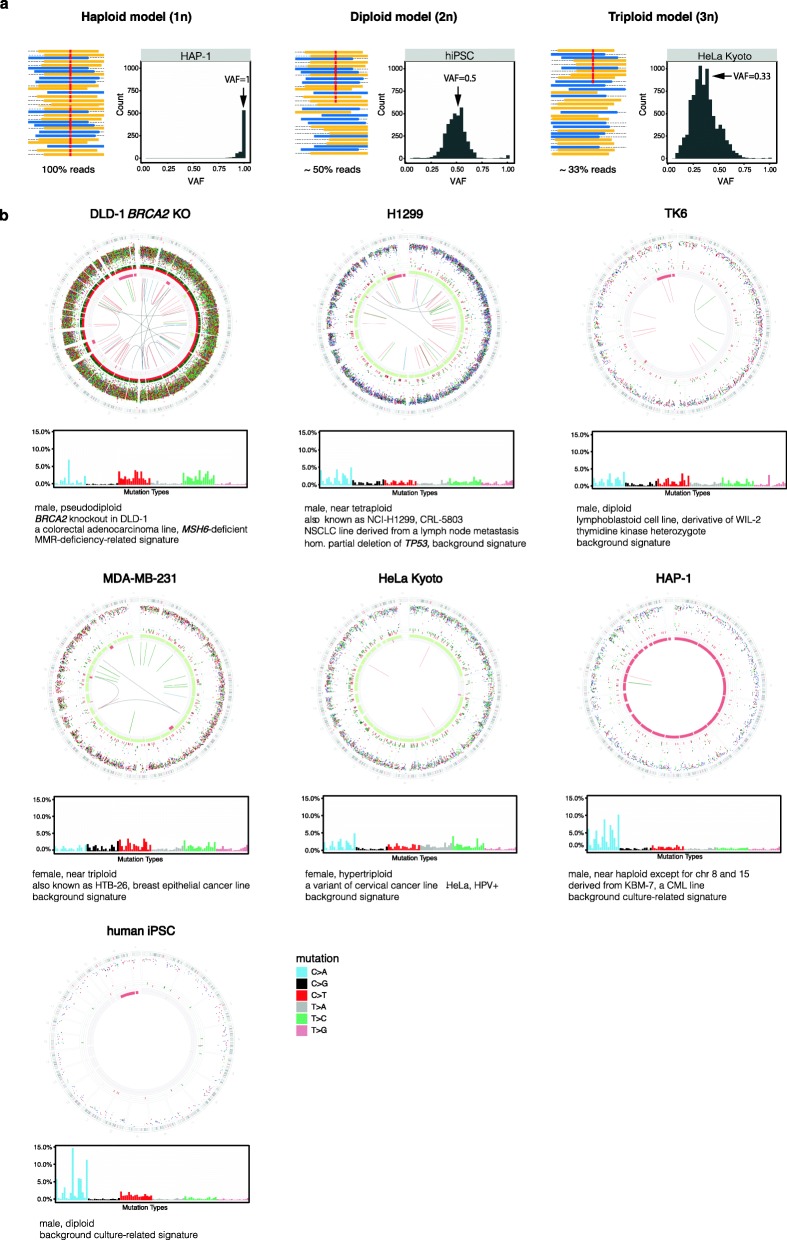
Choice of cellular model systems. **a** Effect of cellular ploidy on the proportion of NGS reads representing variant alleles and on variant allele fraction (VAF) distribution. Blue and yellow lines joined by a dotted line represent forward and reverse reads, respectively (only parts of pair-end reads are shown). Horizontal red lines represent the position of a variant on the sequencing reads. **b** Genome plots and 96-bar plots representing mutational profiles of different cell lines. Shown from the outermost rings (genome plots) moving inwards are (i) the karyotypic ideogram; (ii) base substitutions, plotted as rainfall plots (log_10_ (intermutation distance) on the radial axis; dot colour: blue, C>A; black, C>G; red, C>T; grey, T>A; green, T>C; pink, T>G); (iii) insertions shown as short green lines; (iv) deletions shown as short red lines; (v) major (green blocks, gain) and minor (red blocks, loss) copy number alleles; and (vi) rearrangements shown as central lines (green, tandem duplications; red, deletions). Mutation burdens in the genome plots are non-representative here as different cell lines have had different lengths of time in culture. CML, chronic myelogenous leukaemia; hiPSC, human induced pluripotent stem cell ; HPV, human papillomavirus; NSCLC, non-small cell lung carcinoma

Ideally, a cellular model with a diploid (or haploid) genome should be sought. Gene editing a haploid or diploid model is more efficient than editing a polyploid model. Having a lower ploidy also results in greater proportional representation of mutations that arise in next-generation sequencing reads, increasing the sensitivity of mutation detection (Fig. 1a). In a hyper-triploid (3n+) cell line like HeLa, newly acquired somatic mutations may be present in one allele out of three, reported in ~ 33% of reads. By contrast, a diploid line would report mutations with greater certainty, in ~ 50% of reads. There is also the consideration of sequencing cost: to achieve comparable sensitivity of mutation detection, sequencing a haploid line or an experimental model with a smaller genome (e.g. yeast) would be more affordable than sequencing a diploid or polyploid human model system.

Non-cancerous lines may be preferable because they are less physiologically abnormal. They may have “stemness” properties such as in induced pluripotent stem cells (iPSCs) and embryonic stem cells (ESCs), or they may have tissue-specific properties such as tissue-derived organoids and retinal pigment epithelial (RPE1) cells. Non-cancerous lines are, however, more challenging to grow in culture and less tolerant of manipulation. They may be less likely to manifest mutational signatures because DNA repair and checkpoint pathways are functioning appropriately (or more so), and are thus less permissive for revealing mutagenesis. For example, *TP53*-intact iPSCs do not tolerate double-strand breaks (DSBs), tend to undergo apoptosis quickly, and do not generate rearrangements patterns. Stem cells may also have other physiological properties that effectively protect them in their “stemness” state, and this could have consequences on the likely manifestation of DNA damage, for example, biochemical inactivation of certain drugs because of higher expression of metabolic enzymes or enhanced drug efflux because of higher expression of multifunctional efflux transporters [15, 16].

By contrast, cancer cell lines thrive in culture and will more likely yield patterns of genomic instability. Nonetheless, they often have severely abnormal physiological backgrounds, a multitude of pathway abnormalities acquired in vivo and ex vivo, and thereby carry highly disarrayed genomes (Fig. 1b). Cancer cell lines derived from patients with relapsed disease are likely to be even more pathophysiologically awry, with effects on mutational outcome [17]. Such lines will have been exposed to a multitude of natural and iatrogenic insults, may have highly disordered genomes, and been subjected to extensive rounds of selection pressure promoting evolvability within the cell population. This could culminate in increased mutagenesis. Counterintuitively, it could also result in reduced mutagenesis if the physiological compensation to overcome selective pressure leads to physiological shifts that tend to suppress DNA damage [17]. The chosen biological model must also be amenable to clonal expansion following single-cell bottlenecking, and here, cancer cell lines tend to fare better than immortalised normal cells.

Additionally, it is crucial to know whether a cell line model already carries intrinsic, on-going mutagenesis because profound intrinsic mutational patterns could drown out the signals being sought. For instance, the colorectal cancer cell line DLD-1 *BRCA2*^KO^—albeit a bona fide mismatch repair-deficient cell line—is often used as an HR-deficient model due to its *BRCA2* knockout (KO) status. WGS of DLD-1 *BRCA2*^KO^ cells, however, shows marked mutational signatures associated with MMR deficiency (Fig. 1b), likely to obscure the subtler signals from *BRCA2* deficiency or anything else that would be engineered into this model system.

Some mutagenesis experiments may require an on-going mutational signature in order to dissect mechanisms of mutation formation. In that instance, it would be valuable to identify cell lines with an on-going signature of interest, and engineer perturbations to see how the signature deviates from its intrinsic state.

In a proof-of-principle study, we demonstrated the feasibility of recreating cancer mutational signatures in vitro using CRISPR-Cas9 gene editing in a near-haploid cell model system, HAP-1 [7]. This cell line has a very low level of intrinsic mutagenesis, mainly associated with cell culture that results in C>A mutations (Fig. 1b), thought to be caused by oxidative stress [18, 19]. In contrast to cancer cell lines such as H1299, MDA-MB-231, HeLa which have higher average ploidy, HAP-1 is also near-haploid—thus, sequencing was more affordable (only sequenced to 15×). That it has a propensity to revert to a diploid state is however recognised, and regular inspection must be implemented to detect such a situation for long-term maintenance in culture [20, 21].

**Fig. 2:**
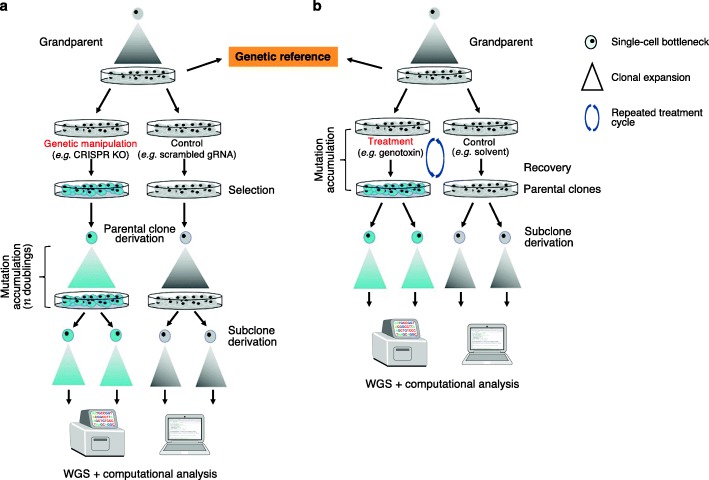
Experimental designs. **a** An example of a genetic manipulation experiment to link specific gene edit to mutational patterns. Note that upon parental clone derivation, mutations might accumulate over several cellular generations to reveal mutational patterns. The number of doublings (*n*) required for mutation accumulation is gene- and/or model system-specific. **b** An example of a genotoxin treatment experiment to link specific genotoxin exposure to mutational patterns. Here, mutations accumulate as a result of treatment; treated cells (and controls) are effectively the parental clones. Repeated cycles of treatment following recovery may help amplify the signals by increasing the mutation burden. It is imperative to sequence grandparental clones as the *normal* genetic reference. The background mutagenesis can be determined through the control subclones

To investigate mutagenesis in cellular models, an isogenic “grandparental” sample of the cellular model system of choice should be used as the genetic reference from which all parental clones are derived (Fig. 2). Here, parental clones refer to a single-cell derived colony that has been through a particular experimental process, such as gene editing of a particular locus and then selected for the desired feature (e.g. knockout of gene X) or exposure to a genotoxin with recovery post-exposure.

### Genetic manipulation to generate gene-edited parental clones

Most mutational signatures extracted from cancers are associated with either exogenous mutagen exposures (e.g. signature 7 with UV; signature 22 with aristolochic acid I) or dysregulation of key DNA repair/replication genes (e.g. signature 3 with *BRCA1*/*BRCA2* mutations; signature 10 with *POLE* mutations). One of the most straightforward experimental strategies to explore mutational signatures is therefore to knock out a gene of interest, knock in an activating mutation, or overexpress a particular protein, to see if the genetic manipulation instigates mutagenesis.

To generate knockouts, aliquots of cells are exposed to reagents designed to target genes of interest. Negative editing controls should be included in parallel experiments, in which cells receive no manipulations or non-targeting versions of the gRNA. These controls are informative of background and/or intrinsic mutagenesis inherent to the cell line models. Following enrichment of edited cells by selection markers—most commonly in the form of a fluorescence reporter or an antibiotic resistance gene—multiple single-cell edited clones can be isolated and screened. Those carrying desired mutations in the given gene are designated parental clones (Fig. 2a). In scenarios where an empty vector or a scrambled gRNA control is unavailable, a clone that has been through targeting for gene knockouts but has nevertheless survived without biallelic alteration in the given gene could be used as the “wildtype” control.

In a knockout or knockdown experiment, loss or downregulation of the proposed target can be ascertained through the confirmation of protein loss via western blot or mass spectrometry [22]. Functional assays may be performed—for example, RAD51 formation assay for an HR gene knockout, although the directness of these relationships is often assumed.

Once verified, parental clones are cultured for a designated period to allow for mutation accumulation (Fig. 2a). The time required for mutation accumulation may vary between targeted genes and would need to be determined empirically, striking a balance between the time in culture and the cost of the experiment.

Some gene KOs may not produce discernible mutational signatures owing to low rates of mutagenesis under standard cell culture conditions. Artificially inducing DNA damage such as with cisplatin could magnify mutagenesis beyond its intrinsic baseline mutation rate, increasing the likelihood of uncovering a signature. It could, however, produce a non-physiological pattern because of the exogenous stressor, and thus, interpretation of such patterns should be made with the experimental set-up in mind. Using alternative isogenic models that are more permissive for mutagenesis (e.g. mouse embryonic fibroblasts, chicken DT40 lymphoblast cell line, or cancer cell lines) may increase mutation rates [23–28]. However, using different cell-based systems of different species or with different genetic backgrounds could result in diverse mutational signatures and must be taken into consideration when interpreting data. For example, cyclophosphamide and cisplatin signatures in DT40 are different from those observed in human cellular models [28].

### Genotoxin exposure

To interrogate mutational signatures associated with exposure to environmental mutagens or genotoxins, aliquots of an isogenic cell line are treated with the chemical in question (Fig. 2b). Appropriate solvent controls must be considered. For example, cisplatin stock should be constituted in 0.9% NaCl instead of DMSO as the latter could cause ligand displacement and reduce cytotoxic effects of the compound. Furthermore, when treated with cisplatin, cells should be treated with 0.9% NaCl in a parallel control experiment to detect potential mutagenesis incurred by the solvent. In addition, many compounds are pro-mutagens and require cytochrome P450-mediated metabolic activation into DNA-reactive intermediates to exert DNA damaging effects. Accordingly, when using these mutagens, the experiments could be performed in the absence and presence of an exogenous metabolising system such as the S9 rodent liver-derived metabolic enzyme mixture with the mutagen of interest [8].

Treating cells with either a chronic, low-dose or punctuated, high-dose exposure becomes another point to consider. Typically, half-maximal inhibitory concentration (i.e. IC50 dose) of a compound is used as a starting point. In a previous study, we treated human iPSCs with 79 environmental mutagens using doses corresponding to either the IC50s or IC80s of the compounds for 2 to 24 h, followed by single-cell bottleneck subcloning upon treatment recovery [8]. Notably, these cells were only treated once. Repeated cycles of treatment following recovery could conceivably increase mutation burden. Selection of resistant clones might, however, develop during a chronic experimental process and needs to be considered particularly if no signatures are seen when they were expected.

Following treatment, successful DNA damage induction is most commonly confirmed via immunofluorescence staining or western blotting of DNA damage response proteins. Routinely, gH2Ax, phospho-p53, phospho-p21, pRPA, pATM, and pATR are used as markers for confirming DNA damage and DNA damage response (DDR) signalling. Nevertheless, successful DNA damage induction does not always correlate with mutagenic outcome; the reverse is also true [8]. For instance, formaldehyde treatment does not induce detectable DDR signalling in human iPSC cells but is associated with a mutation pattern, whereas acetaldehyde and acrylamide are able to elicit DDR, but do not produce detectable mutation patterns [8]. Thus, DDR induction does not necessarily predict mutagenesis.

### Mutation accumulation phase

To detect mutation patterns in experiments involving gene editing, the parental clone is grown under standard culture conditions, for an empirically determined number of cell doublings to allow for mutations to accrue at a steady state (Fig. 2a). For accurate estimation of mutation rate per cellular division, proliferation assays could be considered to determine the doubling time of the parental clones. For experiments involving exposure to environmental mutagens or protein overexpression, mutations accumulate as a consequence of the exposure. Cells are usually given time to recover post-exposure.

At the end of the mutation accumulation phase, the parental cell population will have increased in size and be polyclonal, meaning that each cell will carry its own set of mutations, although some very early, shared mutations may be present. Thus, it is necessary to perform a single-cell subcloning step at the end of mutation accumulation in the parental clone (Fig. 2).

### Single-cell bottleneck

Following the expansion of parental clones, multiple single-cell subclones can be derived through limiting dilution or fluorescence-activated cell sorting with a flow cytometer (FACS). This single-cell bottleneck is necessary to permit detecting mutagenesis that has arisen in individual cells in the parental population using current sequencing technologies. Multiple subclones are required for each gene edit or treatment condition, and serve as technical replicates, permitting assessment of the consistency of mutational signatures between different subclones. Generally, we find that sequencing more replicate subclones (≥ 3) provides greater discriminatory power to discern mutational signatures than increasing mutation accumulation time in culture.

To ensure subclones are derived from a single cell, cellular isolation can be monitored real-time using live-cell analysis systems such as an IncuCyte. If a live-cell stain (e.g. Calcein) is used, single-cell sorted culture plates can be imaged with fluorescence microscopy to confirm that each well only contains a single cell.

Subclones are expanded in culture until sufficient cell numbers are reached for WGS without PCR amplification. For customary 30-fold WGS, approximately 250–500 ng of genomic DNA is required. A diploid human cell contains roughly 6 pg of genomic DNA. Thus, approximately 100,000 cells are needed for whole-genome sequencing a sample.

## Computational analysis

WGS is performed on single-cell derived subclones following mutation accumulation. The grandparental sample is used as the genetic reference to subtract variants that have arisen prior to the grandparental sample and to subtract all shared variants in the parental samples. This allows detection of new (de novo) mutations that arise as a consequence of experimental manipulation. Alternatively, parental clones can also be used as a reference, although this would incur extra sequencing costs as many additional parental clones would need to be sequenced.

After obtaining WGS, short-read sequences of all samples are independently aligned to the reference genome. All classes of somatic mutations are called in subclones against the parental/grandparental clone. In the following section, we demonstrate the use of WGS data for assessing the quality of and relationships between experimental samples and for determining experimentally derived mutational signatures.

### Quality control

**Fig. 3:**
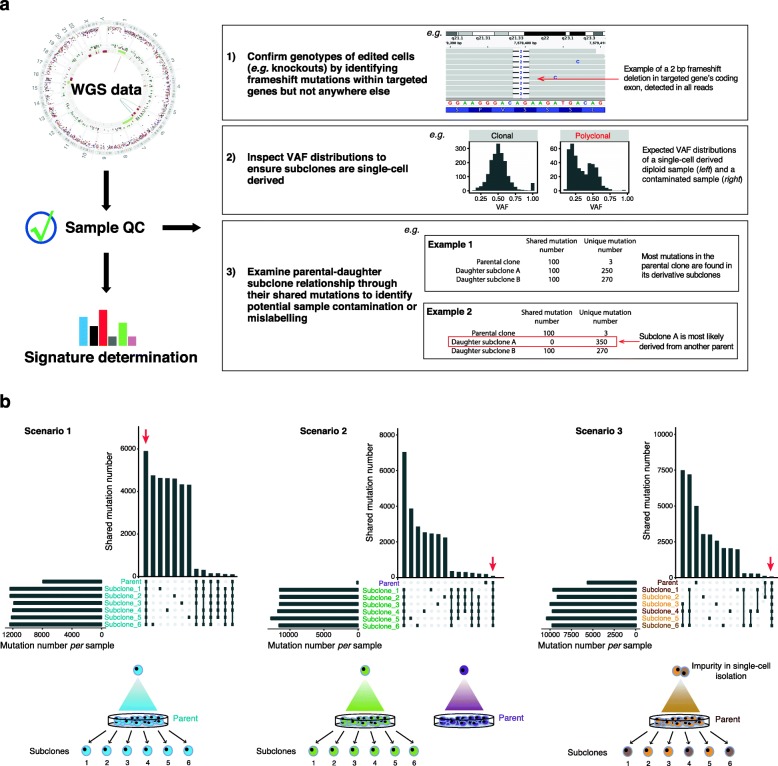
Quality control (QC) of WGS data. **a** Using WGS data to perform QC on experimental samples. **b** Using shared variants between parental and daughter subclones to identify relationships between samples. Three scenarios are presented. The upper panel shows two histograms per experiment, with the total number of mutations per sample (horizontal histogram) and shared mutations between samples (vertical histogram, in decreasing order). Subclones and parental clones for each scenario are also noted. Subclones that share mutations are dotted *black* and connected with a line. The lower panel depicts hypothetical experiments. A red arrow indicates shared mutations between daughter subclones and parental clones. Scenario 1: all subclones are correctly derived from the same parental clone as they share mutations among themselves and with their designated parent, and also have unique mutations. Scenario 2: all subclones are derived from the same parental clone as they share high numbers of mutations among themselves but not with the sequenced parent. In this example, an incorrect parental clone (purple) has been sequenced. Scenario 3: subclones are derived from a mixed parental population. Not all subclones share a high number of mutations among themselves and with their sequenced parent. Subclones 1, 4, and 6 are likely from one lineage; subclones 2, 3, and 5 are from a different lineage. Note that clonal expansion for isolated daughter subclones is not depicted in the diagram for simplicity

To ensure the observed mutational signatures are correctly associated with the proposed experimental conditions, several essential quality control steps may be implemented (Fig. 3a).

First, WGS offers a rapid, straightforward way of checking the genotype of an edited cell line. Successful CRISPR-Cas9 editing of a gene should result in short indels near the gRNA-targeted sequence for a knockout.

Similarly, off-target effects can be detected by explicitly seeking frameshift indels and large structural variants in the rest of the genome. Potential off-target sites for a given gRNA sequence can be queried by using relevant bioinformatic tools, e.g. COSMID (http://crispr.bme.gatech.edu) [29] and WGE (https://www.sanger.ac.uk/htgt/wge/) [30]. Unintended edits might affect a critical gene and result in unexpected mutator phenotypes.

Moreover, it is important to ensure that the model system remains stable and does not develop overt malignant potential through the experimental process. As a rule of thumb, chromosome copy number in all subclones should remain relatively unchanged from their parent unless the treatment or edits are expected to generate copy number variation. Evidence of selection, including clonal and subclonal mutations in all DNA repair genes and *TP53*, and driver amplifications should remain absent from all samples. To ensure that experimentally generated signatures are not a consequence of another genetic defect acquired during culture or treatment, mutations in coding sequences that could influence mutational outcomes should be sought.

Second, variant allele fractions (VAFs) can be used to ascertain whether subclones were derived from single cells. For a single-cell derived sample, all acquired mutations should have VAFs of ~ 0.5 in a diploid model because they are present on one of two possible alleles in a heterozygous state (Fig. 1a). Likewise, for haploid and triploid cells, the VAFs are expected to be normally distributed around 1 and 0.33, respectively. Deviation of VAF distribution from the expected may indicate impurity of single-cell isolation (Fig. 3a). Critically, polyclonal or mosaic subclones often show lower average VAFs and falsely elevated mutation burdens, resulting in an overestimation of mutation numbers. Including these samples in the quantitative analysis will likely confound the estimation of mutation rate and burden associated with a particular experimental condition. Nevertheless, polyclonality most often does not alter the mutational profile of subclones, as the patterns may be qualitatively identical even if the quantitative burden of mutations is inaccurate.

Lastly, the likelihood of laboratory errors increases when multiple experimental conditions are investigated simultaneously. To uncover laboratory mix-ups, relationships between parental clones and their respective subclones can be inspected to detect potential mislabelling of subclones. As all subclones are originally derived from their parental clones, all mutations detected in parental clones should be present in their respective subclones, but not in subclones derived from other parents. Based on this genetic concept of relatedness, surveying shared mutations among all samples would enable the identification of mislabelled samples (Fig. 3b) [31].

### Signature channels

Each mutation type (substitution, double substitution, indel, rearrangement) has its distinct set of channels that are used to define signatures. While it would be ideal to have identical channels for experimental data and cancer-derived data, this is not always possible because the burden of mutagenesis can vary greatly between experimental model systems. The ratio of mutations to signature channels is important to consider: too many channels for low yield of mutations will dilute any signal; likewise, too few channels may not offer the resolution required for deriving biological insights. Substitution channels of experimental models tend to be identical to the ones used for cancers. Indel and rearrangement channels tend to be collapsed into fewer channels. To make a comparison with cancer-derived signatures, it is more effective to collapse cancer-derived signatures into the same channels as the experiments rather than stretching the experimental channels to suit the cancer channels.

Substitution channels mirror that which is customarily used in literature. Sequence context immediately 5′ and 3′ to each mutated base is taken into consideration. Since there are 6 classes of base substitution (C>A, C>G, C>T, T>A, T>C, T>G) and 16 possible sequence contexts for each mutated base (5′ A, C, G, or T and 3′ A, C, G, or T), there are 96 possible channels for substitution signature. Double substitutions are two adjacent bases that are mutated, indicating the existence of commonly occurring mutagenic events that cause substitution mutation at neighbouring bases. Double substitution signatures can be defined by 78 strand-agnostic combinations [8, 32]. Here, the 5′ and 3′ sequence contexts are not commonly considered because it creates (4 × 78 × 4 = 1248) too many channels for the yield of double substitutions typically seen in a sample (often < 5 in untreated samples).

Channels for small indels (< 100 bp) generally incorporate the class (deletion versus insertion), motif CG/TA content, and size (1 bp or larger), as well as the nature of flanking sequence at the indel junction: repeat-mediated indels resulting from replication strand slippage or microhomology-mediated indels formed during the repair of DNA double-strand breaks, or none. If polynucleotide repeats flank the motif, the length of the repetitive sequence is also often considered. In some instances, the variation of indel classifications might be insightful in revealing the underlying mutagenesis patterns. For example, for mutagens that are known to affect particular nucleotide preferentially, it might be valuable to extend the indel classification to consider the effect of sequence context [8].

Rearrangement signatures are broadly categorised based on four types of rearrangements, namely tandem duplications, deletions, inversions, and translocations, with further consideration of sizes of the rearranged fragments [7].

### An analytical framework to identify mutational signatures

**Fig. 4:**
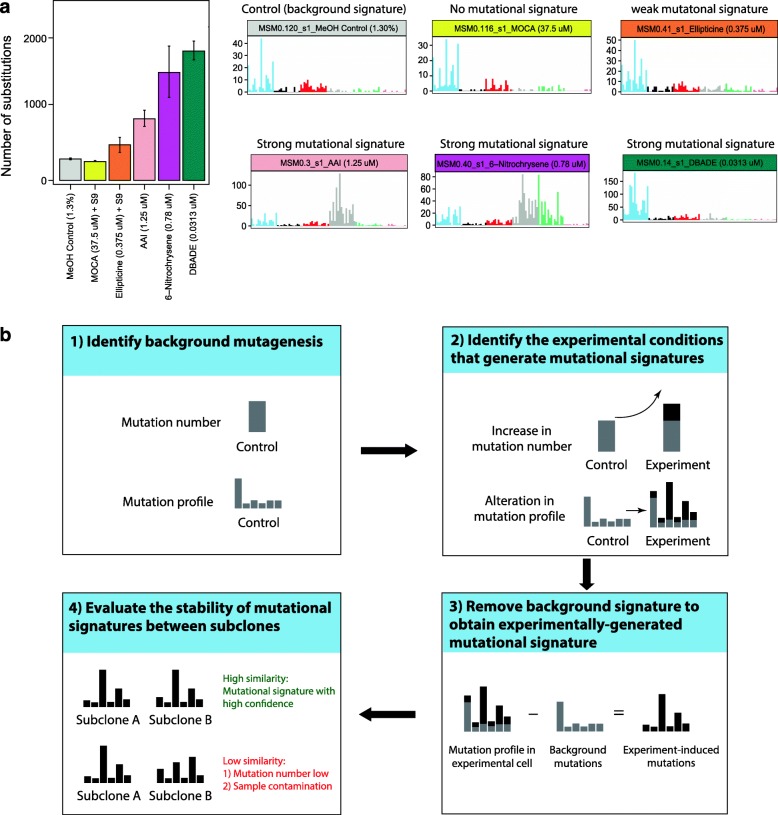
Principles of extracting mutational signatures from experimental samples. **a** Mutation burdens and profiles of human iPSCs treated with different environmental mutagens. Treatments that generate a mutational signature typically show increased mutation burdens and/or altered mutational profiles in the subclones compared to control (background). Note that effect sizes vary for different perturbations. **b** Determining experimentally generated mutational signatures

By comparing the mutational burdens and profiles of experimental subclones with controls, experimental conditions that effectively produce signatures can be identified. The determination of experimentally generated mutational signatures may vary depending on experimental settings, but a general workflow encompasses: (1) identifying background/intrinsic signatures in the chosen cellular system; (2) detecting a quantitative difference in mutation counts between experimental subclones and controls, as well as a qualitative difference in the mutational spectra between experimental subclones and controls; (3) subtracting background/intrinsic signatures to obtain experimentally associated signatures; and (4) evaluating the stability of extracted mutational signatures (Fig. 4).

Pervasive intrinsic signatures may be distinctive in different cell lines. Growing cells in culture also contributes substantial DNA damage that results in particular patterns (Fig. 1b). These two potential sources of background mutagenesis are not negligible; thus, it is necessary to identify and subtract them to determine experimentally generated mutational signatures. In practice, the averaged mutation burden and profile of control subclones can be used to represent the background or intrinsic mutagenesis of the chosen cellular system.

**Fig. 5:**
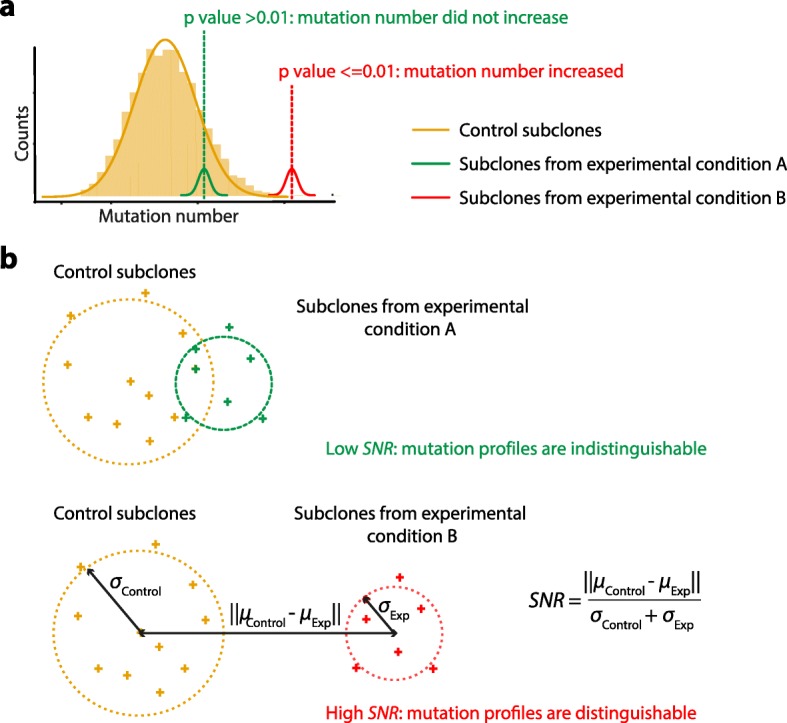
Computational characterisation of experimentally generated mutational signatures. **a** Determination of quantitative difference (i.e. mutation number increase) between experimentally generated subclones and controls through a permutation test based on the distribution of baseline mutation burden in control subclones (orange). A *p* value ≤ 0.01 indicates significantly different mutation burden for experimentally generated subclones (red). **b** Schematic illustration of the distinction of mutational spectra between control and experimental subclones using the *signal-to-noise* ratio (*SNR*). Here, ***μ***_***Control***_ and ***μ***_***Exp***_ denote the means of the mutational profiles of control subclones and experimental subclones, respectively; ***σ***_***Control***_ and ***σ***_***Exp***_ denote the standard deviations of the mutation profiles of control and experimental subclones, respectively. In this example, subclones of experimental condition B can be more confidently separated from the control subclones

The mutational profile of experimental cells is a linear combination of the mutational signature of background mutagenesis and the pertinent experimental manipulation. In principle, if a particular manipulation, whether mutagen treatment or gene edit, generates mutational signatures, one would expect additional mutagenesis above background mutagenesis (Fig. 4). To determine whether there is a significant quantitative increase of mutation numbers in experimental subclones compared to control, bootstrap resampling techniques can be used to construct an “expected” distribution of mutation burdens of control subclones. The likelihood (*p* value) of observing a significantly different mutation burden for experimentally generated subclones can thus be calculated through a permutation test (Fig. 5a).

To ascertain whether there are qualitative differences in mutation profile between experimental subclones and controls, the distinction between mutation profiles can be measured by the *signal-to-nois*e ratio (*SNR*) (Fig. 5b). The Euclidean distance between the mutational profiles of experimental versus control subclones defines the “signal”, while the variability of mutation profiles among subclones defines the “noise” parameter. A large *SNR* value indicates that the difference of mutational profiles between experimental subclones and controls is sufficiently distinguishable from their noises, and therefore, the experiment-associated signature may be separated from the background signature with relative ease. If there is inadequate number of controls for constructing a prior distribution, alternative methods including clustering approaches (e.g. tSNE or contrastive PCA) can be used to identify treated subclones that are distinct from controls. Notably, the number of subclones per experiment and the burden of mutation associated with each experiment are critical to the robustness of the results.

The experiment-associated mutational signature can then be obtained by subtracting the background mutational signature from the mutational profile of treated subclones (Fig. 4b). To do so, each experimental subclone is bootstrapped to generate a distribution of mutation numbers for each signature channel. Based on this distribution, the upper and lower boundaries (99% confidence interval, CI) of mutation numbers for each channel can be calculated. Likewise, a bootstrapped background signature profile can also be generated using the averaged mutation profile and mutation counts. This background can then be subtracted from the centroid of the bootstrapped experimental subclone profiles. This may result in negative values for some channels. However, as long as the numbers fall within the 99% CI of the channels, negative values can be set to zero. Otherwise, the initial background mutation burden has to be reduced.

Ideally, mutational signatures extracted from subclones of the same parental clone should be consistent (Fig. 4b). However, variation may be observed among subclones, particularly when the experiments only incur low mutation burden. The stability of a mutational signature can be reported by calculating the cosine similarity between signatures extracted from subclones. Higher cosine similarity (e.g. > 0.9) lends confidence to the accuracy of extracted mutational signatures.

## Discussion and perspective

As an increasing number of mutational signatures in cancers are being brought to light, studies offering experimental validation have also emerged.

### Association, not causation

There remains a need for some caution in interpretation, even of experimental data. A particular perturbation such as treatment with a chemical, for example, 5-fluorouracil (5-FU) may produce a signature that we recognise [12]. In this case, signature 17, characterised by T>G mutations, widely reported in cancers, of hitherto unknown aetiology. It would, however, not necessarily follow that 5-FU directly causes signature 17. Signature 17 is observed across a broad spectrum of primary tumours that have never been treated with 5-FU and arises spontaneously in untreated mouse embryonic fibroblasts [33–36]. It is far more likely that 5-FU is one of many compounds or physiological stressors of the cell, which, in order to survive, requires a physiological adaptation that results in this hypermutator signature phenotype. In other words, the signature is a secondary, indirect effect of the treatment [37, 38]. These possibilities must be taken into consideration when interpreting signature data, regardless of whether experimental or cancer-derived. As an interesting example, the current COSMIC signature 11, characterised by C>T transitions, was previously attributed to temozolomide because the signature was enriched in tumours of patients that had been treated with this alkylating agent [2]. However, systematic studies using the family of alkylating agents on human IPSCs suggest that the signature of temozolomide is defined by T>C mutations. Another alkylating compound, 1,2-DMH, is instead similar to signature 11 [8, 39].

### Fitting of a priori signatures

Attributing aetiologies to mutational signatures is not as straightforward as may superficially seem. Supervised fitting of signatures could lead to falsely suggested relationships. When we take a set of allegedly “known signatures” and ask the question which of those signatures are present in a new dataset, this process, called “fitting”, is purely mathematical. Presented with 20 potential signatures, the algorithm will do its best to fit all 20 signatures to the data, regardless of whether they are biologically present or not. Thus, presenting signatures that may not be present in a sample but asking the algorithm to fit it to the best of its ability could result in reporting of biological processes that are not present in the sample.

A particularly notorious example is the finding of the “smoking signature” or signature 4 in a variety of different tumour types, even when it is unlikely that tobacco carcinogens could reach said tissue (e.g. prostate). That is because signature 4 is dominated by C>A/G>T transversions and many other signatures also have similar C>A/G>T mutations. The fitting algorithm invokes signature 4 in tissues that have C>A/G>T mutations because it is such a strong phenotype.

Fitting other previously known signatures on experimental data would incur similar risks.

### Using cosine similarity

Mathematically, cosine similarity measures the similarity between two vectors, in this case, the resemblance between two multichannel mutation profiles. However, it does not measure similarity equally across all signatures, working best for sparsely populated signatures that have prominent features (i.e. prominent peaks), and less effectively for flatter, nondescript profiles. Cosine similarity is also not a linear metric—a measure of 0.8, for example, does not imply a high level of correlation. While a measure of 0.99, by contrast, does imply a high level of correlation, it does not mean that they are the same or caused by the same mechanisms. Likewise, the same gene defect or exposure can cause slightly different mutational signatures in different tissues, cautioning against the blind reliance on this metric to assess the similarity between signatures.

### Signatures and aetiologies do not necessarily have 1-to-1 mappings

Some experiments can induce multiple signatures per treatment or knockout. If these signatures arise in different classes, they are immediately interpretable as distinct signatures of different classes. However, if a gene defect produces multiple mutational signatures of the same mutation class, it will not be possible to distinguish them from each other instantly. Additional genetic or physiological stressors may be required to separate two signatures of the same mutation class.

Disparate mutational processes can also produce the same mutational signature outcome. For example, a classical T>A mutation signature that has been associated with aristolochic acid I is nearly identical to the T>A signature induced by Dibenzo[a]pyrene Diol-epoxide (DBPDE), a polycyclic aromatic hydrocarbon that is present in tobacco smoke [8]. These disparate compounds likely converge on the genome in the same way, producing an adduct on adenine that results in a similar outcome. This surprising, humbling result is one that underscores the reason why we cannot simply assume that we understand the full picture based on performing correlative genomic analyses alone. Fundamentally, a 1-to-1 mapping of one gene to one signature or one mutagen to one signature is unlikely to be the norm.

### Signal sizes

The mutation burden generated by different environmental mutagens and different gene knockouts is highly variable. In general, mutagenesis is more pronounced in experiments where external genotoxins are introduced. Notably, many mutagens or gene knockouts do not produce a detectable increase in mutation burden in experimental systems; their signal sizes can be much smaller than observed in human cancers. Several possible reasons might account for this. First, the cellular system of choice may have a genetic background that suppresses DNA damage. Second, excessive or lethal DNA damage might cause apoptosis in normal cells, e.g. *TP53*-intact iPSCs do not produce rearrangement signatures. Third, the culture time and proliferation rate of the cell might affect the rate of mutation accumulation and therefore the signal size. Fourth, there is genetic redundancy of DNA repair in the cell. As a result, some DNA repair gene knockouts may not produce a direct mutational consequence. Fifth, some DNA repair pathways mainly target damage caused by external environmental mutagens, e.g. xeroderma pigmentosum (XP) genes of nucleotide excision repair are involved in repairing UV-induced cyclobutane pyrimidine dimers (CPDs). In normal cell culture condition (no UV radiation), XP gene knockouts indeed do not generate mutational signatures.

### Caution in using experimentally generated signatures

Environmental exposures that were the earliest to be associated with patterns in human cancers, well before the advent of whole-genome sequencing such as the signatures associated with tobacco, aristolochic acid, and ultraviolet light, are precisely the experimental treatments with the largest signals. They are orders of magnitude higher in mutagenicity compared to many other mutagens and hence were readily detected in many different experimental models historically. We observed smaller signals from exposures that have weaker DNA damaging impact.

In seeking new “environmental causes of cancer”, we must do so with some caution: Just because we now know the signatures associated with these other agents, does not mean that we can and should use all of these signatures in an a priori way to seek out new causes of cancer in all future cancer datasets. Some thoughtful consideration is required. As mentioned previously, when using a set of a priori signatures, this purely mathematical step is designed to seek out all possible suggested signatures in the dataset, regardless of whether they are genuinely biologically there or not. Thus, we caution the (mis) use of experimentally generated (or any) mutational signatures—poorly considered use of signatures during the fitting step could result in mistakenly interpreting the presence of an environmental mutagen when it is not in a new dataset. Indeed, mis-assigning the presence of an occupational mutagen, for example, could lead to legal claims that are inappropriate.

### Future directions in understanding mechanism

Future studies to explore mutagenesis by inducing specific types of DNA damage in selective DNA repair defective genetic backgrounds represent an attractive avenue to fine-tune our understanding of and to gain further insights into the mechanisms of mutagenesis. To achieve that, direct, genome-wide unbiased and specific measurement of the DNA lesions and their repair is required. For example, by coupling Damage-seq with XR-seq for cisplatin damage [40–42] or DSBCapture seq [43] with whole-genome sequencing for DSBs, one could map precisely where the damage occurs in the genome and chart how cells differentially repair or misrepair the induced damage in different parts of the genome. Dissecting these mechanisms will help us understand the regional heterogeneity in damage sensitivity and the accessibility and efficacy of DNA repair machinery.

Decreased sequencing cost and technical advances in single-cell WGS, as well as long-read sequencing technologies (e.g. PacBio sequencing), will likely transform the field. Long-read sequencing could uncover more and resolve previously understudied large and complex structural variants [44]; single-cell WGS would allow tens and hundreds of cells to be profiled in a single experiment, hence offering more statistical power while at the same time simplifying the experiments by circumnavigating the need for single-cell bottlenecking. Currently, single-cell WGS data still suffer from high levels of noise and artefact variants introduced during whole-genome amplification and cell lysis process [4]. When more single-cell WGS data become available, such artefactual signatures may be better defined and used for filtering out false-positive mutations.

We hope the guidelines presented here could help streamline the design and analysis of future studies.

## Supplementary information

**Additional file 1.** Review history.
