# Supplementary material for: Mutational signatures: experimental design and analytical framework
Source: Genome Biol. 2020 Feb 14;21:37. doi: 10.1186/s13059-020-1951-5 (PMC7023746; doi:10.1186/s13059-020-1951-5)
Supplement: Supplementary file 1 — Additional file 1. Review history. [file 13059_2020_1951_MOESM1_ESM.docx]

Review History

First round of review

Reviewer 1

Is the topic of the article timely and of interest to a wide range of readers?

Yes

Is the article well written and presented in a logical way?

Yes

Do the authors **cover the relevant literature in an accurate and balanced way?**

Yes

**Do the authors provide a useful synthesis of the topic?**

Yes

**Do the authors provide insightful discussion of the future directions for the field?**

Yes: The authors present a valuable guide for experimentally identifying mutational signature causes

**Do any tables, figures or boxes that are present enhance the manuscript?**

Yes

**Would the manuscript benefit from any additional tables, figures or boxes?**

Yes

**Comments to author:**

Koh, Zou, and Nik-Zainal present a valuable guide for experimentally identifying mutational signature causes. I have only minor suggestions for improvement as outlined below.

1.     It would be helpful to briefly delineate some reasons why mutations may not occur in iPS cells upon exposure to environmental mutagens. For example, ALDH expression inactivates certain drugs and tends to be higher in stem cells, and stem cells also tend to have higher levels of drug efflux pumps. The authors hint at this ("…DNA repair and checkpoint pathways are functioning appropriately (or more so)…") but it would be valuable to provide additional details.

2.     Similarly, can the authors speculate on why the reported temozolomide signature differs in patients vs. cell culture (PMID 30236628)? Are there any lessons readers can take from this example as they plan experiments to test for mutational signatures?

3.     In the section "Choice of cellular model system" it would be helpful to mention that many cancer cell lines derive from relapsed patients and thus are more likely to have subclones with drug resistance mechanisms that inhibit mutagenesis (drug efflux, etc.). This is hinted at later ("Selection of resistant clones might, however, develop during a chronic experimental process…") but the pitfalls of using relapsed cell models specifically (discussed in PMID 31697823) could be mentioned, and would reinforce the value of single-cell cloning before the experiment begins as the authors show in Figure 2.

4.     When discussing alternative culture systems ("e.g. mouse embryonic fibroblasts") it would be helpful to alert readers that other species' mutational signatures can be quite different from human signatures. For example, PMID 27161042 shows cisplatin and cyclophosphamide signatures in chicken cells that do not match these drugs' signatures in humans, based on the authors' recent Cell paper (Kucab et al.) and PMID 29632087. This information will help readers be cautious about using models from species distantly related to the index species.

5.     Figure 3 shows a helpful approach for identifying sample mismatches. The most important comparison between panels (a) and (b) appears to be the set of variants present in all subclones (1-6) plus parental cells, which is high in (a) and low in (b). However, it took this reviewer some time to realize this since the mutation clusters are ordered differently in the two panels (that cluster is left-most in panel (a), but right-most in panel (b)). It would be helpful if the ordering could be kept consistent or if other means were used to highlight to the reader which comparisons are the most important.

6.     I would suggest removing the term "novel" from the first sentence of the abstract as mutational signature analysis has become mainstream in recent years.

**Reviewer 2**

**Is the topic of the article timely and of interest to a wide range of readers?**

Yes

**Is the article well written and presented in a logical way?**

Yes

**Do the authors cover the relevant literature in an accurate and balanced way?**

Yes

**Do the authors provide a useful synthesis of the topic?**

Yes

**Do the authors provide insightful discussion of the future directions for the field?**

Yes:The authors provide in-depth discussion about application and future directions of mutational signatures.

**Do any tables, figures or boxes that are present enhance the manuscript?**

No: Some figures are not appropriately organized. Figures should be improved.

**Would the manuscript benefit from any additional tables, figures or boxes?**

Yes

**Comments to author:**

In this review paper, entitled "Mutational signatures: experimental design and analytical framework", Koh and the authors provided a comprehensive guideline about the experimental designs, pitfalls, analysis workflows to discover or relate aetiologies to mutational signatures. Particularly, the detailed description of interrogating mutagen signatures by practical experimental setting (e.g., exposure to mutagen, single cell bottleneck, quality control and computational analysis) would be helpful many researchers understand how mutational signatures are developed and annotated, and even plan their own analyses. Overall, this is a good review paper. However, some parts of the paper, especially figures and main texts can be improved. Detailed comments are like below:

Major comments:
1. Although this paper provides general and essential information about mutational signatures, the authors spend most of the main manuscript on experimental details, which makes this paper more like a protocol paper. This paper would draw general interests from readers if the authors give more insights besides the experimental details such as the current status of mutational signatures and annotated aetiologies, what have been done by these discoveries, and why we need more efforts on this field. Especially, introduction is too brief and does not give sufficient information about the background of this paper. For example, the authors stated "There are differences in how these studies were performed, how data were processed, analysed and interpreted with different algorithms." at the end of second paragraph of introduction with 12 references.
2. Some figures are not well organized or referred from the main text:
- The legends of Figure 1a and 1b are switched
- The lines and circus plots in the raindrop plots in Figure 1b is hard to read.  
- Figure 3 is referred after Figure 4a.
- The compositions of Figure 4 and 5 do not seem optimally organized. Figure 4b is more closely related to items in Figure 5, as they are referred together in the main text.
- Figure 5a is more like a text not figure. And it has not referred independently from the paper. Also, I am not sure if Figure 5 is well composed to describe "Analytical framework to identify mutational signatures" as all the items deal with minor issues not the 'identification of signatures'
3. It would be better to discuss about the error signatures (e.g., sequencing artifact signatures) such as their effects on analysis.

**Authors Response**

**Point-by-point responses to the reviewers’ comments:**

*We would like to thank the reviewers for the favourable reviews.*
Reviewer #1: Koh, Zou, and Nik-Zainal present a valuable guide for experimentally identifying mutational signature causes. I have only minor suggestions for improvement as outlined below.
Thank you for the favourable review. We endeavoured to address the comments below.

1. It would be helpful to briefly delineate some reasons why mutations may not occur in iPS cells upon exposure to environmental mutagens. For example, ALDH expression inactivates certain drugs and tends to be higher in stem cells, and stem cells also tend to have higher levels of drug efflux pumps. The authors hint at this ("…DNA repair and checkpoint pathways are functioning appropriately (or more so)…") but it would be valuable to provide additional details.
*Response: Thank you. This has been added bearing the word limit in mind.
We have added the following:
In line 84: Stem cells may also have other physiological properties, that effectively protect them in their “stemness” state and this could have consequences on the likely manifestation of DNA damage, for example, biochemical inactivation of certain drugs because of higher expression of metabolic enzymes or enhanced drug efflux because of higher expression of multifunctional efflux transporters [15, 16].
Additionally, under the section “Signal sizes” we have added more reasons why mutational signatures may not be detected in certain experimental models so far. Lines 457-468.*

2. Similarly, can the authors speculate on why the reported temozolomide signature differs in patients vs. cell culture (PMID 30236628)? Are there any lessons readers can take from this example as they plan experiments to test for mutational signatures?
*Response: Thank you. We have added specific references to this in line 402 to 407:
“As an interesting example, the current COSMIC signature 11, characterized by C>T transitions was previously attribute to temozolomide because the signature was enriched in brain tumours of patients that had been treated with this alkylating agent. However, systematic studies using the family of alkylating agents on human IPSCs suggest that the signature of temozolomide is defined by T>C mutations. Another alkylating compound, 1,2-DMH is instead similar to Signature 11.”*

3. In the section "Choice of cellular model system" it would be helpful to mention that many cancer cell lines derive from relapsed patients and thus are more likely to have subclones with drug resistance mechanisms that inhibit mutagenesis (drug efflux, etc.). This is hinted at later ("Selection of resistant clones might, however, develop during a chronic experimental process…") but the pitfalls of using relapsed cell models specifically (discussed in PMID 31697823) could be mentioned, and would reinforce the value of single-cell cloning before the experiment begins as the authors show in Figure 2.
*Response: Indeed, we thank the reviewer for this insightful example and have included the following sentences (line 91 to 99):
“Cancer cell lines derived from patients with relapsed disease are likely to be even more pathophysiologically awry, with consequences on mutational outcome. Such lines will have been exposed to a multitude of natural and iatrogenic insults, may have highly disordered genomes and been subjected to extensive rounds of selection pressure promoting evolvability within the cell population. This could culminate in increased mutagenesis. Counter-intuitively, it could also result in reduced mutagenesis if the physiological compensation to overcome selective pressure leads to physiological shifts that tend to suppress DNA damage.”*

4. When discussing alternative culture systems ("e.g. mouse embryonic fibroblasts") it would be helpful to alert readers that other species' mutational signatures can be quite different from human signatures. For example, PMID 27161042 shows cisplatin and cyclophosphamide signatures in chicken cells that do not match these drugs' signatures in humans, based on the authors' recent Cell paper (Kucab et al.) and PMID 29632087. This information will help readers be cautious about using models from species distantly related to the index species.
*Response: Agreed. This is important. Thank you for highlighting it.
We have:
• Included the addition of “e.g. mouse embryonic fibroblasts, chicken DT40 lymphoblast cell line or cancer cell lines” in line 166 page 4
• And added “of different species or” in line 167
• And added references.
• Also added in line 169: “For example, cyclophosphamide and cisplatin signatures in DT40 are different to those observed in human cellular models.”*

5. Figure 3 shows a helpful approach for identifying sample mismatches. The most important comparison between panels (a) and (b) appears to be the set of variants present in all subclones (1-6) plus parental cells, which is high in (a) and low in (b). However, it took this reviewer some time to realize this since the mutation clusters are ordered differently in the two panels (that cluster is left-most in panel (a), but right-most in panel (b)). It would be helpful if the ordering could be kept consistent or if other means were used to highlight to the reader which comparisons are the most important.
*Response: Agreed. Thank you for pointing this out. Figure 3 has been revised (merged with previous Figure 4). And as reviewer #1 kindly pointed out, “The most important comparison between panels (a) and (b) appears to be the set of variants present in all subclones (1-6) plus parental cells, which is high in (a) and low in (b).”, we added to the revised Figure 3b a set of red arrows on top of the bars that indicate the shared variants between sequenced subclones and parental clones. To make it clearer, we also added “Shared mutations between daughter subclones and parental clones are indicated by red arrow” in line 550, and also added “in decreasing order” in line 548 to indicate that the clustering (vertical histogram) is ordered by decreasing counts of shared variants.*

6. I would suggest removing the term "novel" from the first sentence of the abstract as mutational signature analysis has become mainstream in recent years.
*Response: Yes of course, this is now done (line 24). Thank you.*

Reviewer #2: In this review paper, entitled "Mutational signatures: experimental design and analytical framework", Koh and the authors provided a comprehensive guideline about the experimental designs, pitfalls, analysis workflows to discover or relate aetiologies to mutational signatures. Particularly, the detailed description of interrogating mutagen signatures by practical experimental setting (e.g., exposure to mutagen, single cell bottleneck, quality control and computational analysis) would be helpful many researchers understand how mutational signatures are developed and annotated, and even plan their own analyses. Overall, this is a good review paper. However, some parts of the paper, especially figures and main texts can be improved. Detailed comments are like below:
*Thank you.*

Major comments:
1. Although this paper provides general and essential information about mutational signatures, the authors spend most of the main manuscript on experimental details, which makes this paper more like a protocol paper. This paper would draw general interests from readers if the authors give more insights besides the experimental details such as the current status of mutational signatures and annotated aetiologies, what have been done by these discoveries, and why we need more efforts on this field. Especially, introduction is too brief and does not give sufficient information about the background of this paper. For example, the authors stated "There are differences in how these studies were performed, how data were processed, analysed and interpreted with different algorithms." at the end of second paragraph of introduction with 12 references.
*Response: The purpose of this manuscript is exactly to provide a reference of how to conduct these experiments and analyse them. We agree with the reviewer that reviewing the field with respect to “the current status of mutational signatures and annotated aetiologies” will be of greater interest in general, but that is beyond the scope of this practical exercise. The reviewer’s point is being addressed in an alternative general review manuscript.*

2. Some figures are not well organized or referred from the main text:
- The legends of Figure 1a and 1b are switched
*Response: Thank you. This has been corrected.*


- The lines and circus plots in the raindrop plots in Figure 1b is hard to read.
*Response: Thank you. We have rearranged and enlarged the circos plots. The intention of showing the circos plots of different cell line models here is to convey the overall visual contrast that different cell lines exhibit different background/ intrinsic mutagenesis. For clarity, we have added to the figure legend (line 526): “Mutation burdens are non-representative here as the different cell-lines have had different lengths of time in culture”.*

- Figure 3 is referred after Figure 4a.
*Response: Thank you. We agree with the reviewer’s comment. We have merged Figure 3 with Figure 4a, see revised Figure 3 and Figure 4. In-text citations have been updated accordingly.*

- The compositions of Figure 4 and 5 do not seem optimally organized. Figure 4b is more closely related to items in Figure 5, as they are referred together in the main text.
*Response: Thank you. Figure 4b is now revised. See new Figure 4 and 5.*

- Figure 5a is more like a text not figure. And it has not referred independently from the paper. Also, I am not sure if Figure 5 is well composed to describe "Analytical framework to identify mutational signatures" as all the items deal with minor issues not the 'identification of signatures'
*Response: Figure 5a is now removed.*

3. It would be better to discuss about the error signatures (e.g., sequencing artifact signatures) such as their effects on analysis.
*This is not an unreasonable issue that the reviewer raises. However, it is beyond the scope of this review to address this point.
Sequencing artefacts that are detected in general mutational signature analyses in cancers may be seen because of systematic artefacts of library-preparation, sequencing chemistry, variability in quality of mutation-calling, and mapping abnormalities. Some of these are recognisable while others may be new. It is possible for current mutational signatures analytical processes to pick these up, the difficulty is in interpreting whether they are a new signature or simply an artefact. We could discuss and debate this area extensively but it is not strictly relevant to the purpose of this article.
Sequencing artefacts are not an issue for these sorts of experiments which are conducted in a highly-controlled manner, with controls performed in parallel.*

**Second round of review**

**Reviewer 2**

The author successfully answered most of my previous questions. Figure quality has been improved and textual errors were corrected.
